# Effects of L-Valine Supplementation in Low-Nitrogen Diets on Rumen Fermentation Parameters, Predicted Methane Emissions Production, and Microbial Communities In Vitro

**DOI:** 10.3390/ani16071049

**Published:** 2026-03-30

**Authors:** Chuang Li, Yang Liu, Tianao Yang, Zhanyuan Chen, Guotuo Jiang, Kailun Yang, Mengzhi Wang

**Affiliations:** 1 College of Animal Science, Xinjiang Agricultural University, Urumqi 830091, China; chuangliyzu@163.com; 2 Laboratory of Metabolic Manipulation of Herbivorous Animal Nutrition, College of Animal Science and Technology, Yangzhou University, Yangzhou 225009, China; mz120231608@stu.yzu.edu.cn (Y.L.); yta18943927398@163.com (T.Y.); 3 Research Quality Control Center, Jiangsu Sanyi Animal Nutrition Technology Co., Ltd., Xuzhou 221300, China; chenzhanyuan202309@163.com (Z.C.); jgt600@126.com (G.J.); 4 State Key Laboratory of Sheep Genetic Improvement and Healthy Production, Xinjiang Academy of Agricultural Reclamation Sciences, Shihezi 832000, China

**Keywords:** low-nitrogen diet, L-valine, in vitro, rumen, fermentation parameters, microbial community structure

## Abstract

Few studies have investigated the effects of L-valine (L-Val) supplementation on in vitro rumen fermentation parameters and methane (CH_4_) production in low-nitrogen diets for ruminants. Therefore, we examined the impact of L-Val supplementation in low-protein diets on in vitro rumen fermentation parameters, CH_4_ production, and microbial community structure. Two crude protein (CP) levels and 4 L-Val levels were tested as follows: CON group (Control group with 14.05% CP), LD group (low-nitrogen diets with 11.26% CP), LVA group (LD group + 0.25% L-Val), LVB group (LD group + 0.5% L-Val), LVC group (LD group + 0.75% L-Val), and LVD group (LD group + 1% L-Val). The experiment was conducted at five time points (2 h, 4 h, 8 h, 12 h, 24 h), with three replicates per treatment at each time point. Results indicated the following: (1) Fermentation pH decreased overall with time; at each time point, the LVB group exhibited the highest pH, significantly higher than the LD, LVC, and LVD groups (*p* < 0.05). (2) Ammonia nitrogen (NH_3_-N) concentration increased over time, with LVA~LVD groups showing higher levels than the LD group at 24 h, while showing no difference compared to the CON group (*p* > 0.05). (3) Microbial protein (MCP) trends aligned with NH_3_-N, with the LVB group exhibiting higher MCP than the LD group, while showing no difference compared to the CON group (*p* > 0.05). (4) Compared to the LD group, adding 0.5~1% L-Val increased acetic acid, total VFA (TVFA), and isobutyric acid concentrations at 4 h, 8 h, and 24 h fermentation (*p* < 0.05). (5) The LVB group exhibited higher proportions of protozoa and *Fibrobacter succinogenes* (*F. succinogenes*) compared to the LD group (*p* < 0.05). The proportion of *F. succinogenes* showed no significant difference from the CON group (*p* > 0.05), while the proportion of *Butyrivibrio fibrisolvens* (*B. fibrisolvens*) decreased when L-Val addition exceeded 0.5%. (6) Correlation analysis revealed positive correlation between protozoa and TVFA (R = 0.512, *p* = 0.030). Isobutyric acid showed positive correlations with protozoa, *B. fibrisolvens*, and *F. succinogenes* (*p* < 0.05). In summary, under the present experimental conditions, the addition of 0.5% L-Val to a low-nitrogen diet did not affect predicted CH_4_ production, but improved other in vitro rumen fermentation parameters, including acetate, isobutyrate and MCP. Meanwhile, it favored the growth and proliferation of the fibrolytic bacteria (*B. fibrisolvens* and *F. succinogenes*). This provides a theoretical basis for the rational formulation of low-nitrogen diets for sheep.

## Simple Summary

Ruminants exhibit low-nitrogen utilization efficiency, resulting in substantial nitrogen (N) excretion that pollutes the environment. To mitigate this issue, one of the most effective measures is reducing dietary protein levels. Valine (Val), a branched-chain amino acid, is both an essential amino acid for livestock and poultry and a limiting amino acid for rumen microorganisms. This study aimed to evaluate the effects of L-Val supplementation on rumen fermentation parameters, CH_4_ emissions, and microbial community structure under low-nitrogen dietary conditions. Results indicated that supplementing 0.5% L-Val to a low-nitrogen diet improved rumen fermentation and promoted the growth and proliferation of fiber-degrading bacteria.

## 1. Introduction

Dietary protein levels are recognized as a core determinant of growth, development, and health in livestock and poultry [1,2]. A large proportion of protein feed ingredients are obtained from crops that compete with human food supplies, such as soybeans, other oilseeds, and grains [3]. Therefore, against the backdrop of a growing global population and increasing demand for animal products, prudent management of these limiting feeds is crucial for the sustainable development of the livestock industry [4]. Moreover, they represent the most expensive and efficient components in livestock feed formulations [5]. Balanced N intake helps control N pollution, thereby contributing to environmental and economic sustainability [6]. Excessive N intake in ruminants increases N excretion, leading to undesirable nitrate leaching into groundwater and ammonia emissions into the atmosphere [7]. Although N is vital for all organisms, ruminants exhibit relatively low production efficiency in N utilization [8].

Reducing N levels in diets helps mitigate environmental pollution caused by excessive N emissions. Appropriate supplementation of amino acids in diets can promote animal growth, thereby improving feed efficiency and productivity to a certain extent [9,10]. Compared to monogastric animals, research on this approach in ruminants is limited, primarily constrained by limiting amino acids such as lysine (Lys), methionine (Met), tryptophan (Try), and threonine (Thr), often overlooking the importance of branched-chain amino acids (BCAAs; Leucine (Leu), Isoleucine (Ile), and Valine (Val)). BCAAs constitute approximately 20% of total amino acids in muscle, 35% of essential amino acids, and 50% of essential amino acids in milk protein [11,12,13]. Consequently, BCAAs supply is critical for protein turnover efficiency. Due to their unique functions and metabolic characteristics, BCAAs have garnered increasing attention from ruminant nutrition researchers. Research indicates that supplementing with branched-chain amino acids can promote fermentation by rumen bacteria [14]. Research by Wang [15] and Wang [16] demonstrated that removing BCAAs from in vitro culture media containing histidine, Lys, Met, and BCAAs most significantly impacted microbial protein yield, limiting rumen microbial growth. Among the three BCAAs, L-Val has been shown to play a particularly critical role in rumen microbial growth and nitrogen utilization. Nolte [17] investigated the effects of BCAAs on N utilization efficiency in the rumen of Rambouillet rams. Results showed that removing any three BCAAs or L-Val from the infused amino acid mixture significantly reduced N utilization efficiency, with L-Val having the most pronounced effect.

L-Val functions as a non-protein biomolecule in the body’s nutritional metabolism (lipid metabolism, glucose metabolism), stress responses, and tissue development [18]. L-Val improves lactation function in breeding stock and is considered one of the limiting amino acids in dairy cattle, poultry, and swine feeds [19,20]. L-Val metabolism contributes to maintaining the stability and health of the rumen microbial community by promoting protein synthesis and energy production [21]. Furthermore, L-Val serves as a growth factor for rumen cellulolytic bacteria, typically exerting positive effects on rumen microbial fermentation and feed efficiency [22]. To our knowledge, existing research on L-Val in ruminants has primarily focused on dairy ruminants, with in-depth investigations into its role in milk synthesis mechanisms [19,23]. However, studies examining L-Val’s effects on rumen fermentation in meat sheep under low-nitrogen diets remain scarce.

Therefore, this experiment aims to investigate the impact of different L-Val supplementation levels on in vitro rumen fermentation in meat sheep fed low-nitrogen diets. We hypothesized that dietary supplementation with an appropriate level of L-Val could improve in vitro rumen fermentation parameters and alter the rumen microbial community under a low-nitrogen diet.

## 2. Materials and Methods

### 2.1. Materials

The L-Val used in the experiment was purchased from Hulunbuir Northeast Fufeng Biotechnology Co., Ltd. (Hulunbuir, China) with an effective content greater than or equal to 98%.

### 2.2. Experimental Design

This experiment comprised six treatment groups: the CON group (base diet CP level 14.05%), the LD group (base diet CP level 11.26%), and the LVA, LVB, LVC, and LVD groups, which were based on the LD group diet, supplemented with 0.25%, 0.5%, 0.75%, and 1.0% L-Val, respectively. The nutritional levels of the control group substrate were formulated according to NYT 816-2021 [24]. The CP level in the LD group was reduced by approximately 20% relative to the CON group. Metabolic energy levels were maintained as consistent as possible between the two groups. The composition and nutrient levels of the fermentation substrate are shown in Table 1. Each group underwent 2 h, 4 h, 8 h, 12 h, and 24 h gradient fermentation treatments, with three biological replicates at each fermentation time point.

### 2.3. In Vitro Rumen Fermentation

The fermentation broth consisted of buffer solution and fresh rumen fluid. The rumen fluid used in the experiment was collected from the ventral sac of the rumen before morning feeding from five sheep fitted with permanent rumen fistulas at the Animal Breeding Base of Yangzhou University. Rumen fluid buffer was prepared using artificial saliva salts and reducing agents according to the method of Menke et al. [25]. The chemical reagents contained in the buffer solution were NaHCO_3_ (8.75 g/L), NH_4_HCO_3_ (1.00 g/L), Na_2_HPO_4_ (1.43 g/L), KH_2_PO_4_ (1.55 g/L), MgSO_4_·7H_2_O (0.1581 g/L), Na_2_S (0.52 g/L), MnCl_2_·4H_2_O (0.015 g/L), CoCl_2_·6H_2_O (0.002 g/L), FeCl_3_·6H_2_O (0.012 g/L), CaCl_2_·2H_2_O (0.017 g/L), and resazurin (1.25 mg/L).

A 0.5 g sample of the mixed substrate was weighed into individual 100 mL culture bottles. Five time points (2 h, 4 h, 8 h, 12 h, and 24 h) were set for each group, with three replicates at each time point. Then, 25 mL of fresh rumen fluid was mixed thoroughly with 50 mL of saliva buffer solution (1:2) and injected into the culture bottles. CO_2_ was immediately introduced for 3–5 s; the bottles were then capped and incubated in a water bath constant-temperature shaker (SHA-A, Jintan Hengfeng Yiji Manufacturing Co., Ltd. Changzhou, China). After incubation started, fermentation was terminated at each corresponding time point using an ice-water bath. The culture medium was collected after filtration through four layers of sterile gauze, divided into four 5 mL centrifuge tubes, and stored at −80 °C for subsequent analysis of microbial content, ammonia nitrogen (NH_3_-N), microbial crude protein (MCP), and concentrations of volatile fatty acids (VFAs), including acetic acid, propionic acid, butyric acid, iso-butyric acid, valeric acid, and isovaleric acid.

### 2.4. Determination of Fermentation Parameters in Culture Medium

The pH of the culture medium was measured immediately after fermentation termination at each time point using a pH meter. The NH_3_-N content in the culture medium was determined according to the method of Zhang et al. [26] (phenol-sodium hypochlorite colorimetric method). VFAs concentrations were determined using the internal standard method on a gas chromatograph (GC-14B, Shimadzu Corporation, Kyoto, Japan) [27]. Rumen fluid samples were centrifuged at 12,000× *g* for 15 min. The supernatant was then collected, and the microbial crude protein (MCP) concentration in rumen fluid was determined with reference to the Coomassie brilliant blue method [28].

### 2.5. Methane Production Forecast

Based on the fact that 80% of hydrogen produced from VFA metabolism is utilized for CH_4_ synthesis, this study employs the CH_4_ prediction model established by Moss et al. [29] to forecast rumen CH_4_ yield. The CH_4_ yield prediction model is as follows:

CH_4_ = 0.45 × acetic acid − 0.275 × propionic acid + 0.4 × butyric acid

In the equation, acetic acid, propionic acid, and butyric acid are expressed as molar ratios. CH_4_ represents the estimated CH_4_ yield from volatile fatty acids, measured in mmol/mol.

### 2.6. Determination of Relative Abundance of Microorganisms in Culture Medium

#### 2.6.1. DNA Extraction

Total DNA was extracted from the culture medium using a fecal genomic DNA extraction kit (Tiangen Biochemical Technology Co., Ltd., Beijing, China). After thawing 1 mL of fermentation broth at 4 °C, extraction was performed according to the kit instructions. Following DNA extraction, the concentration and purity of the total DNA were assessed at OD 260/280 and OD 260/OD 230 using a micro-volume spectrophotometer (NanoDrop-1000, Thermo Fisher Scientific, Waltham, MA, USA). DNA fragment size was verified via 0.7% agarose gel electrophoresis. The DNA was then stored at −80 °C for future use.

#### 2.6.2. Primer Sequences

Total bacteria, protozoa, *Ruminococcus amylophilus* (*R. amylophilus*), *Streptococcus bovis* (*S. bovis*), *F. succinogenes*, *B. fibrisolvens*, and Methanogenus were detected using real-time quantitative PCR. Primer design followed the methodology of Khafipour et al. [30], with detailed primer sequences listed in Table 2. All primers and internal controls were purchased from GenKaiRui Biotechnology Co., Ltd. (Wuhan, China)

#### 2.6.3. PCR Amplification

In real-time quantitative PCR experiments, amplification was performed using a 20 μL reaction system comprising the following components: 2 μL DNA template, 0.8 μL upstream primer, 0.8 μL downstream primer, 0.4 μL Reference Dye I, 10 μL SYBR Premix Ex Taq™ II (TaKaRa Bio, Tokyo, Japan), and 6 μL sterile distilled water.

The amplification program was set as follows: initial denaturation at 95 °C for 5 min, denaturation at 95 °C for 5 s, annealing/extension at 58 °C for 34 s, and a final extension at 72 °C for 30 s, repeated for 40 cycles. The threshold cycle number (Ct) was obtained by analyzing real-time fluorescence data. The relative expression of each gene was normalized to total bacteria as a reference gene, and the quantitative changes of the target bacteria in the experimental group were further calculated. The relative expression levels of target genes were calculated using the 2^−ΔΔCt^ method, with the formula: Relative Expression = 2^−[ΔCt(target)−ΔCt(control)]^ where ΔCt represents the difference in Ct values between the target gene and the reference gene [31].

### 2.7. Data Statistics and Analysis

Preliminary data cleaning was performed using Excel (V2016). Subsequently, one-way ANOVA analysis was conducted using the ANOVA module in IBM SPSS Statistics 25 software, followed by Duncan’s multiple range test for post hoc comparisons. Differences were considered non-significant when *p* > 0.05 and significant when *p* < 0.05. Correlations between microbial parameters and rumen fermentation parameters were calculated using the Cor package in R (V 4.5.1). A correlation existed when *p* < 0.05 and |R| > 0.5 [32], where R represents the Pearson correlation coefficient.

## 3. Results

### 3.1. Effect of L-Valine Supplementation in Low-Nitrogen Diets on the pH of In Vitro Rumen Fermentation Fluid

As shown in Table 3, after 4 h, the LVB group and CON group showed no significant difference (*p* > 0.05) but were higher than the LVD group (*p* < 0.05). After 8 h, the LVB group was higher than the CON group, LD group, and LVD group (*p* < 0.05). After 12 and 24 h of incubation, the LVB group exhibited the highest pH, which was higher than that of the LD, LVC, and LVD groups (*p* < 0.05).

### 3.2. Effect of L-Valine Supplementation in Low-Nitrogen Diets on NH_3_-N In Vitro Rumen Fermentation Broth

As shown in Table 4, the NH_3_-N concentration in the fermentation broth ranged from 6.26 to 12.81, with an increasing trend over time across all groups. After 2 h and 4 h of incubation, the NH_3_-N concentration in the CON group was higher than in the other groups (*p* < 0.05), while no differences were observed among the other groups (*p* > 0.05). After 8 h incubation, the differences between the CON group and the LVA, LVB, and LVD groups were not significant, but the concentration was significantly higher than that in the LD group (*p* < 0.05). After 24 h incubation, the NH_3_-N concentrations in the culture fluids of the LVA, LVB, LVC, and LVD groups did not differ significantly from that in the CON group (*p* > 0.05), but were significantly higher than that in the LD group (*p* < 0.05).

### 3.3. Effect of L-Valine Supplementation in Low-Nitrogen Diets on MCP In Vitro Rumen Fermentation Fluid

As shown in Table 5, after 2 h of culture, the MCP concentration in the CON group culture medium was significantly higher than that in other groups (*p* < 0.05), while no significant differences were observed among the other groups (*p* > 0.05). At 4 h and 8 h, compared with the CON group, the LVB and LVD groups showed no difference (*p* > 0.05), but the MCP concentration in the culture medium of the LD, LVA, and LVC groups was reduced (*p* < 0.05). After 24 h of culture, compared with the CON group, the MCP concentration in the LVB group culture medium was not different from that in the CON group (*p* > 0.05), but was higher than that in the LD group and other groups (*p* < 0.05).

### 3.4. Effects of L-Valine Supplementation in Low-Nitrogen Diets on VFA In Vitro Rumen Fermentation

#### 3.4.1. Effects of L-Val Supplementation in Low-Nitrogen Diets on Short-Chain VFAs In Vitro Rumen Fermentation Broth

As shown in Table 6, for acetic acid, after 4 and 8 h of culture, the concentration difference in the culture medium between the CON group and the LD and LVA groups was not significant (*p* > 0.05), but it was significantly lower than that in the LVB, LVC, and LVD groups (*p* < 0.05). After 12 h of incubation, the acetic acid concentration in the CON group culture medium did not differ significantly from that in the LD group (*p* > 0.05), but was significantly lower than that in the LVD group (*p* < 0.05). After 24 h of culture, the acetic acid concentration in the CON group was not significantly different from that in the LD group (*p* > 0.05), but was significantly lower than that in the LVA, LVB, LVC, and LVD groups (*p* < 0.05). For butyric acid, compared with the CON group, the concentration in the LD group culture medium was significantly reduced (*p* < 0.05), but there was no significant difference compared with the LVA, LVB, and LVC groups (*p* > 0.05). For valeric acid, after 4 h of culture, the concentrations in the LD and LVC groups were not significantly different from the CON group (*p* > 0.05), but were significantly higher than those in the LVA, LVB, and LVD groups (*p* < 0.05). After 8 h of culture, the concentrations of valeric acid in the culture media of the CON, LD, LVC, and LVD groups did not differ significantly (*p* > 0.05), but were significantly higher than those in the LVA group (*p* < 0.05). After 24 h of incubation, the concentration of valeric acid in the culture medium of the LVC group was significantly higher than that in the CON, LD, LVA, and LVB groups (*p* < 0.05). Compared with the CON group, the concentration of isobutyric acid in the culture medium of the LVA, LVB, LVC, and LVD groups was significantly increased after 4 h and 12 h of incubation (*p* < 0.05), but the difference was not significant compared with the LD group (*p* > 0.05). After 8 h of culture, compared with the CON group, the concentration of isobutyric acid in the culture medium of the LVB, LVC, and LVD groups was significantly increased (*p* < 0.05), but the difference with the LD group was not significant (*p* > 0.05). After 24 h of culture, compared with the CON group, the isobutyric acid concentration in the LD group was not significantly different but was significantly lower than that in the LVA, LVB, LVC, and LVD groups, with the highest levels observed in the LVB and LVC groups. Furthermore, the propionic acid and isovaleric acid concentrations in the culture media of all groups did not differ significantly among groups at any times (*p* > 0.05).

#### 3.4.2. Effects of L-Val Supplementation in Low-Protein Diets on TVFA and Acetate/Propionate In Vitro Rumen Fermentation

As shown in Table 7, the concentration of TVFA in the culture medium increased over time across all groups. After 4 h and 24 h of culture, TVFA concentrations in the LVB, LVC, and LVD groups were significantly higher than those in the CON group (*p* < 0.05), while no significant differences were observed compared to the LD and LVA groups (*p* > 0.05). After 8 h of culture, compared with the CON group, the LD group showed no significant difference in TVFA concentration between the LVB, LVC, and LVD groups and the CON group (*p* > 0.05), but was significantly higher than the LD and LVA groups (*p* < 0.05). After 12 h of culture, TVFA levels in the LVA, LVB, LVC, and LVD groups were significantly elevated compared to the CON group (*p* < 0.05), but no significant difference was observed between the CON group and the LD group (*p* > 0.05). In addition, the acetate / propionate ratio did not differ between groups at any culture time point.

### 3.5. Effects of L-Valine Supplementation in Low-Nitrogen Diets on Microorganisms In Vitro Rumen Fermentation Broth

**Figure 1:**
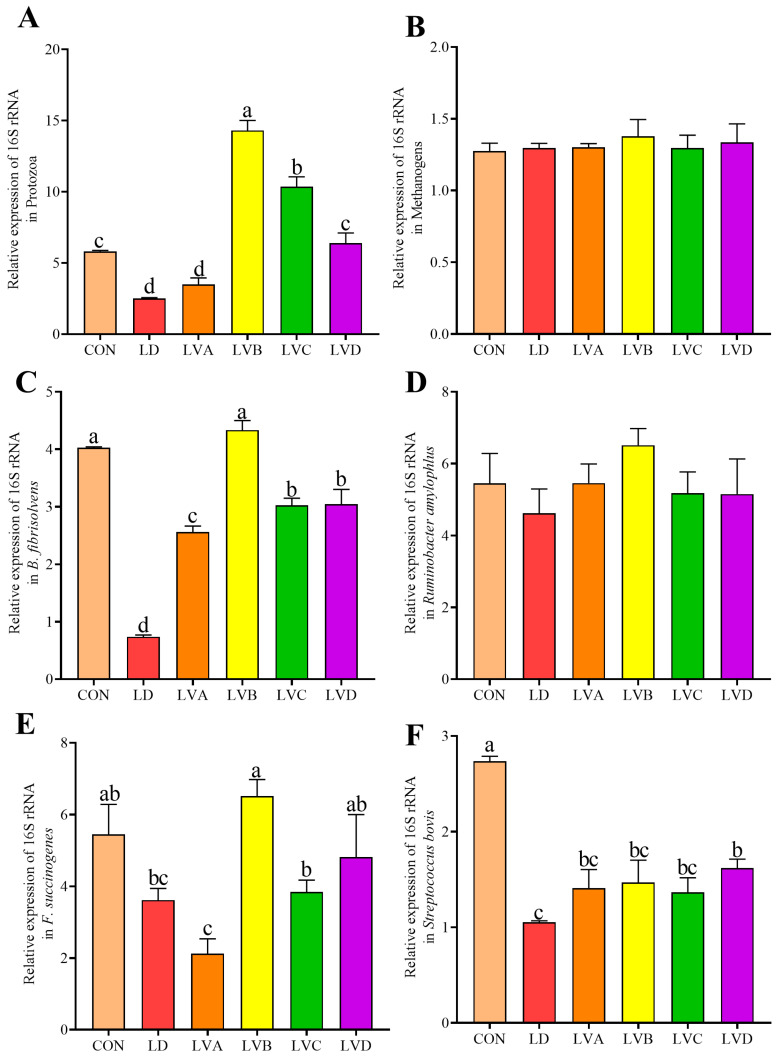
Effects of L-Val supplementation in low-nitrogen diets on microorganisms in vitro rumen fermentation broth. (**A**) Protozoa; (**B**) Methanogens; (**C**) *Butyrivibrio fibrisolvens*; (**D**) *Ruminobacter amylophlus*; (**E**) *Fibrobacter succinogenes*; (**F**) *Streptococcus bovis*. Note: Different letters above the bars indicate significant differences among dietary treatments (*p* < 0.05).

As shown in Figure 1, compared with the CON group, the proportion of protozoa in the culture media of the LVB and LVC groups significantly increased (*p* < 0.05), while it significantly decreased in the LD and LVA groups (*p* < 0.05). Compared with the CON group, the proportion of *B. fibrisolvens* in the culture media of the LD, LVA, LVC, and LVD groups decreased (*p* < 0.05). The proportion of *F. succinogenes* in the LVB and LVD groups was not significantly different from that in the CON group (*p* > 0.05), but was higher than that in the LD and LVA groups (*p* < 0.05). The proportion of *S. bovis* in the CON group culture medium was the highest and significantly higher than that in the other groups (*p* < 0.05).

### 3.6. Effect of L-Valine Supplementation in Low-Nitrogen Diets In Vitro Rumen CH_4_ Production

As shown in Table 8, the CH_4_ production in each culture medium group did not differ significantly at any time points (*p* > 0.05).

### 3.7. Correlation Analysis of Microorganisms and Fermentation Parameters

**Figure 2:**
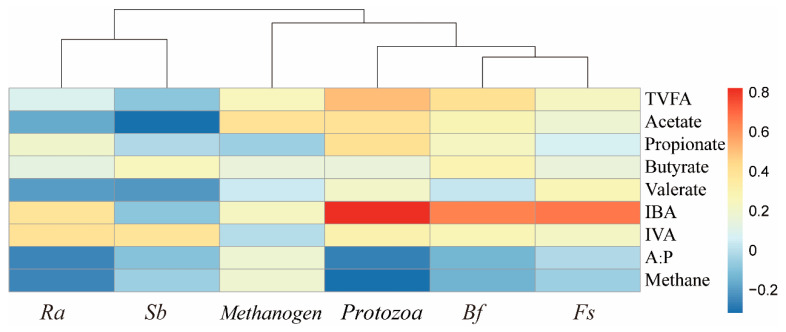
Correlation analysis of microorganisms and rumen fermentation parameters. Ra: *Ruminobacter amylophilus*; Sb: *Streptococcus bovis*; Bf: *Butyrivibrio fibrisolvens*; Fs: *Fibrobacter succinogenes*; IBA: Isobutyric acid; IVA: Isovaleric acid; A:P: Acetate:Propionate; TVFA: Total volatile fatty acid.

The correlation analysis results between microbial and fermentation parameters (Figure 2) indicate that protozoa showed positive correlation with TVFA (R = 0.512, *p* = 0.030). IBA showed positive correlations with protozoa (R = 0.820, *p* < 0.001), *B. fibrisolvens* (R = 0.641, *p* = 0.004), and *F. succinogenes* (R = 0.665, *p* = 0.003).

## 4. Discussion

pH, NH_3_-N, MCP and VFAs are crucial factors affecting rumen fermentation. The normal fluctuation range of ruminal pH is 5.5–7.5, which is crucial for maintaining stable physiological functions [33,34]. When the rumen fluid pH is below 6.2, the growth and reproduction of fibrolytic bacteria will be inhibited [35,36]. In addition, the LVB group maintained the highest pH at all time points and remained above 6.2, which was conducive to microbial proliferation.

NH_3_-N is an intermediate product of rumen microbial decomposition of N-containing substances and can reflect the potential for MCP synthesis and degradation [37]. The optimal concentration of NH_3_-N in rumen fluid ranges from 2.37 to 27.3 mg/dL [38]. In this experiment, the NH_3_-N concentration ranged from 6.26 to 12.81 mg/dL, all falling within the reasonable interval. The NH_3_-N concentration in the CON group was significantly higher than that in the LD group at 2 h, 4 h, 8 h, and 24 h, which might be attributed to the reduced CP level that lowered the NH_3_-N concentration in rumen fluid. As N levels increase, the concentration of NH_3_-N in rumen fluid rises, which may be related to the increase in non-protein N [39], and a similar result was reported by Wu et al. [40]. At the 8 h and 24 h incubation time points, there was no significant difference in NH_3_-N concentration among the LVA, LVB, LVD and CON groups, but these groups exhibited significantly higher concentrations than the LD group. Studies have demonstrated that compared with other amino acids, L-Val has the slowest degradation rate of 0.1–0.14 mM/h. With the extension of incubation time, L-Val is gradually degraded to produce ammonia and nitrogen. On the other hand, L-Val may enhance the N degradation capacity of protein-degrading bacteria, thereby increasing the NH_3_-N concentration. As a synthetic product of NH_3_-N, MCP showed a consistent variation trend with NH_3_-N [41]. The key to the efficient utilization of N in ruminant nutrition lies in the temporal and spatial synchronization between N availability in the rumen and fermentable carbohydrates [42,43]. When the N synchronization is in a good state, NH_3_-N can be timely utilized by microorganisms for MCP synthesis, thereby making the variation trends of MCP and NH_3_-N consistent. In this experiment, the overall variation pattern of MCP was consistent with that of NH_3_-N. In addition, it is worth noting that at 24 h of incubation, there was no significant difference in MCP concentration between the LVB group and the CON group, but the LVB group had a higher MCP concentration than the LD group. This suggested that L-Val supplementation could improve the capacity of converting NH_3_-N to MCP under low-nitrogen dietary conditions. Allison [44] pointed out that isobutyric acid can promote the uptake and utilization of NH_3_-N by rumen bacteria, thereby decreasing the NH_3_-N content in the rumen and increasing microbial protein synthesis.

Carbohydrates in the diet are degraded by rumen microbes to produce VFAs, which serve as crucial energy substrates for the maintenance, growth and development of ruminants. The results of this experiment showed that at 4, 8 and 24 h of fermentation, compared with the LD group, supplementation with 0.5%, 0.75% and 1% L-Val under low-nitrogen conditions significantly increased the molar concentrations of acetic acid, TVFA and isobutyric acid. Acetic acid in the rumen is the primary product of fiber degradation [45]. The significant increase in isobutyric acid concentration might be associated with the degradation of L-Val into isobutyric acid by rumen microbes [46]. In addition, isobutyric acid is essential for the growth and proliferation of fibrolytic bacteria, thereby increasing the concentration of acetic acid [47]. Previous studies have demonstrated that supplementing diets with isobutyric acid increases the abundance of *B. fibrisolvens* and *F. succinogenes* in the rumen of pre- and post-weaning Holstein calves [48]. In addition, 0.5% dietary L-Val significantly increased valeric acid concentration after 24 h of incubation. The increased valeric acid concentration indicates enhanced microbial fermentation of amino acids and improved N turnover [49,50]. In the present study, the increase in valeric acid concentration may be attributed to the incorporation of the carbon skeleton of L-Val into fatty acid synthesis or degradation, ultimately leading to its elevated concentration.

CH_4_ is the end product of carbohydrate metabolism within the rumen and a key component of energy metabolism. The genetic characteristics of ruminant breeds, their physiological stage, and the source and composition of dietary nutrients are primary factors influencing CH_4_ production. Low-nitrogen diets represent an effective strategy for modifying CH_4_ production in ruminants. Lamba et al. [51] observed that increasing the dietary content of rumen-undegradable protein correspondingly reduced CH_4_ production. Shirmohammadi et al. [52] reported that after 24 h of fermentation, the high-protein control group (16.2%) produced 22.05 mL/g DM of CH_4_, while the LD group produced 21.18 mL/g DM, with no significant difference between groups. Kreuzer et al. [53] reported that reducing dietary protein from 150 g/kg to 125 g/kg in dairy cows decreased CH_4_ yield from 6.3% to 5.7%. In this experiment, we fitted CH_4_ production based on acetate, propionate, and butyrate yields. However, it should be emphasized that the classic stoichiometric equation proposed by Moss et al. [29] has obvious limitations in explaining and predicting CH_4_ production in the present study. As verified by Ellis et al. [54], the Moss equation assumes a fixed and excessively high hydrogen recovery rate (90%), whereas the actual hydrogen recovery rate during rumen fermentation is much lower (28.9–56.2% or approximately 80%). This leads to systematic overestimation of CH_4_ production and poor prediction accuracy. Furthermore, the Moss model is overly simplistic and overlooks key biological processes in the rumen, including hydrogen loss, alternative hydrogen sinks, complex microbial interactions, and functional diversity of protozoa. Results indicate no significant difference in CH_4_ production between the LD and CON groups. Furthermore, supplementing the LD group with L-Val did not reduce CH_4_ production, likely due to unchanged methanogenic microorganism populations. Notably, the increase in rumen protozoa did not alter CH_4_ production. Rumen protozoa are involved in methane and acetate production via the generated H_2_ [55,56]. Therefore, in the present study, after L-Val intake, rumen protozoa may have shifted H_2_ from the methanogenic pathway to the acetogenic pathway, thereby increasing acetate concentration without increasing CH_4_ content.

Reducing the N level in the diet significantly decreased the proportion of protozoa, fiber-degrading butyric acid-producing bacteria, and *S. bovis* in the culture medium. Rumen protozoa play a crucial role in rumen fermentation in ruminants. They phagocytose bacteria, inhibit their digestion and absorption of dietary starch, and simultaneously convert dietary fiber and starch into VFAs. This process slows carbohydrate fermentation in the rumen and stabilizes rumen pH [57,58]. Within the rumen microbial community, protozoa constitute 50% of the total microbial biomass [59]. Studies indicate that rumen protozoan abundance and diversity positively correlate with protein levels, consistent with the present findings [60]. *B. fibrosolunticum* plays a vital role in nutrient digestion and absorption in basal diets, contributing to cellulose degradation, protein hydrolysis, and butyrate production during starch breakdown [61]. He et al. [62] found that feeding Holstein bulls a high-protein diet increased the relative abundance of cellulolytic *Bifidobacterium* and *Eubacterium* in the rumen, which are involved in biohydrogenation and cellulose degradation. *S. bovis* is widely present in the rumen as a major protein-degrading bacterium, but its abundance remains low even under high-concentrate diet conditions [63]. In addition, it is worth noting that, as a facultative anaerobe, *S. bovis* has a strong ability to ferment non-fibrous carbohydrates such as soluble starch, and can rapidly degrade starch into lactic acid and VFAs [64,65], which leads to a sharp decrease in ruminal pH [66]. Therefore, *S. bovis* plays a very important role in the study of ruminal acidosis.

## 5. Conclusions

The present study indicates that under buffered in vitro culture conditions with low-nitrogen substrate, supplementation with 0.5% L-Val resulted in higher pH values during fermentation than other groups and improved the conversion efficiency of NH_3_-N to MCP during the late fermentation stage, reaching levels similar to those in the normal nitrogen group. Unfortunately, no positive effect on predicted CH_4_ production was observed. Meanwhile, 0.5% L-Val increased the concentrations of acetate, TVFA and isobutyric acid, and elevated the relative abundances of protozoa and *F. succinogenes* in the culture fluid under low-nitrogen conditions.

Overall, under low-nitrogen conditions, moderate addition of L-Val could exert beneficial effects on in vitro rumen fermentation parameters (without affecting CH_4_) and microbial community structure. Further, in vivo studies are warranted to explore the regulatory effects of L-Val in low-nitrogen diets.

## Figures and Tables

**Table 1:** Substrate composition and nutritional composition of the control group and low-nitrogen group (DM basis).

Items ^1^	CON ^2^	LD ^3^	Items ^4^	CON	LD
Ingredient Composition			Nutritional Composition		
Alfalfa	10.00	10.00	ME MJ/kg	10.84	10.95
Wheat Straw	30.00	30.00	CP (%)	14.05	11.26
Corn Stalk	10.00	10.00	EE (%)	2.16	2.26
Corn	23.25	27.10	NDF (%)	39.90	39.62
Wheat Bran	6.00	6.80	ADF (%)	24.32	24.11
Soybean Meal	10.25	5.90	Ca (%)	1.09	1.09
Chili Pepper Meal	5.00	5.00	P (%)	0.49	0.47
Urea	0.50	0.20			
CaCO_3_	1.00	1.00			
CaHPO_4_	1.00	1.00			
Salt	1.00	1.00			
Premix ^5^	2.00	2.00			

^1^ CaCO_3_ = Calcium Carbonate, CaHPO_4_ = Calcium hydrogen phosphate, ^2^ CON: Control group. The basal diet was fed with a CP level of 14.05%. ^3^ LD: low-nitrogen group, reduce crude protein by approximately 20% based on the baseline diet. ^4^ Metabolizable energy was a calculated value, while the others were measured value. ME = Metabolizable energy, CP = Crude protein, EE = Ether extract, NDF = Neutral detergent fiber, ADF = Acid detergent fiber. ^5^ The premix provided the following nutrient content for one kilogram of diet: Cu 150 mg, Fe 500 mg, Zn 1000 mg, Mn 500 mg, VA 100,000 IU, VE 800 mg, VD3 50,000 IU, niacin 60.00 mg, biotin 0.2 mg.

**Table 2:** Fluorescent quantitative PCR primer sequences for rumen microorganisms.

Items	Primer	Amplified Fragment
Total bacteria	F: GAAGAGTTTGATCATGGCTCAG	352
	R: CTGCTGCCTCCCGTAG	
Protozoon	F: GCTTTCGWTGGTAGTGTATT	234
	R: CTTGCCCTCYAATCGTWCT	
*R. amylophilus*	F: CTGGGGAGCTGCCTGAAT	100
	R: CATCTGAATGCGACTGGTTG	
*S. bovis*	F: TTCCTAGAGATAGGAAGTTTCTTCGG	127
	R: ATGATGGCAACTAACAATAGGGGT	
*F. succinogenes*	F: GTTCGGAATTACTGGGCGTAAA	97
	R: CGCCTGCCCCTGAACTATC	
*B. fibrisolvens*	F: ACCGCATAAGCGCACGGA	65
	R: CGGGTCCATCTTGTACCGATAAAT	
Methanogen	F: GGATTAGATACCCSGGTAGT	232
	R: GTTGARTCCAATTAAACCGCA	

**Table 3:** Effect of L-Val supplementation in low-nitrogen diets on the pH of in vitro rumen fermentation fluid.

Time/h	Treatment ^1^						SEM ^2^	*p*
	CON	LD	LVA	LVB	LVC	LVD		
2	6.88	6.88	7.07	6.79	6.74	6.75	0.051	0.489
4	6.71 ^a^	6.68	6.65	6.73 ^a^	6.63	6.57 ^b^	0.016	0.019
8	6.42 ^b^	6.38 ^b^	6.47	6.57 ^a^	6.45	6.39 ^b^	0.018	0.005
12	6.27 ^ab^	6.06 ^c^	6.34 ^ab^	6.40 ^a^	6.05 ^c^	6.18 ^bc^	0.035	<0.001
24	6.09 ^b^	6.07 ^bc^	6.04 ^bc^	6.29 ^a^	5.98 ^c^	5.98 ^c^	0.027	<0.001

^1^ CON: Control group; LD: Low-nitrogen group; LVA: LD group + 0.25% L-Val; LVB: LD group + 0.5% L-Val; LVC: LD group + 0.75% L-Val; LVD: LD group + 1% L-Val. ^2^ SEM, standard error of the mean. ^a–c^ indicate significant differences between values in the same row at *p* < 0.05.

**Table 4:** Effects of L-Val supplementation in low-nitrogen diets on NH_3_-N in vitro rumen fermentation fluid (mg/dL).

Time/h	Treatment ^1^						SEM ^2^	*p*
	CON	LD	LVA	LVB	LVC	LVD		
2	7.52 ^a^	6.45 ^b^	6.44 ^b^	6.83 ^b^	6.26 ^b^	6.51 ^b^	0.117	0.003
4	7.82 ^a^	6.56 ^bc^	7.07 ^b^	7.08 ^b^	6.91 ^bc^	6.97 ^c^	0.102	0.001
8	8.35 ^ab^	6.89 ^c^	8.61 ^ab^	8.91 ^a^	7.86 ^b^	8.58 ^ab^	0.184	0.001
12	8.58	7.16	8.25	8.32	8.13	8.41	0.182	0.275
24	12.81 ^a^	8.22 ^b^	12.39 ^a^	12.38 ^a^	12.20 ^a^	12.10 ^a^	0.432	0.001

^1^ CON: Control group; LD: Low-nitrogen group; LVA: LD group + 0.25% L-Val; LVB: LD group + 0.5% L-Val; LVC: LD group + 0.75% L-Val; LVD: LD group + 1% L-Val. ^2^ SEM, standard error of the mean. ^a–c^ indicate significant differences between values in the same row at *p* < 0.05.

**Table 5:** The Effect of L-Val supplementation in low-nitrogen diets on MCP in vitro rumen fermentation fluid (mg/mL).

Time/h	Treatment ^1^						SEM ^2^	*p*
	CON	LD	LVA	LVB	LVC	LVD		
2	0.66 ^a^	0.53 ^b^	0.55 ^b^	0.56 ^b^	0.55 ^b^	0.54 ^b^	0.012	0.005
4	0.74 ^a^	0.62 ^c^	0.63 ^bc^	0.69 ^ab^	0.66 ^bc^	0.67 ^abc^	0.012	0.028
8	0.80 ^a^	0.68 ^bc^	0.65 ^c^	0.78 ^ab^	0.63 ^c^	0.78 ^ab^	0.020	0.011
12	0.79	0.71	0.74	0.75	0.72	0.83	0.015	0.103
24	0.91 ^a^	0.79 ^c^	0.85 ^c^	0.89 ^ab^	0.86 ^c^	0.88 ^c^	0.009	<0.001

^1^ CON: Control group; LD: Low-nitrogen group; LVA: LD group + 0.25% L-Val; LVB: LD group + 0.5% L-Val; LVC: LD group + 0.75% L-Val; LVD: LD group + 1% L-Val. ^2^ SEM, standard error of the mean. ^a–c^ indicate significant differences between values in the same row at *p* < 0.05.

**Table 6:** Effects of L-Val supplementation in low-protein diets on VFAs in vitro rumen fermentation (mmoL/L).

Item	Time/h	Treatment ^1^						SEM ^2^	*p*
		CON	LD	LVA	LVB	LVC	LVD		
Acetic acid	2	36.62	36.79	37.68	37.35	37.67	37.51	0.143	0.097
	4	44.02 ^c^	44.26 ^c^	45.27 ^bc^	46.14 ^ab^	45.99 ^ab^	46.81 ^a^	0.288	0.005
	8	46.79 ^c^	46.94 ^c^	47.44 ^bc^	49.19 ^ab^	48.51 ^ab^	48.53 ^a^	0.264	0.011
	12	56.21 ^bc^	55.70 ^c^	57.88 ^ab^	58.06 ^ab^	58.31 ^ab^	58.16 ^a^	0.32	0.027
	24	61.51 ^c^	61.69 ^bc^	63.49 ^ab^	63.90 ^a^	64.06 ^a^	64.23 ^a^	0.338	0.018
Propionic acid	2	11.09	11.94	12.11	11.76	11.88	12.05	0.126	0.191
	4	13.87	14.41	14.47	15.38	14.71	14.86	0.181	0.268
	8	14.29	15.38	14.69	14.76	15.35	15.15	0.148	0.216
	12	16.2	16.48	16.96	16.55	16.03	16.2	0.152	0.608
	24	20.99	21.27	22.07	23.21	22.01	22.41	0.33	0.474
Butyric acid	2	9.59 ^a^	8.29 ^c^	8.94 ^abc^	8.78 ^ab^	9.19 ^abc^	8.51 ^bc^	0.135	0.035
	4	8.83	8.48	8.79	8.56	8.45	9.02	0.109	0.592
	8	10.8	10.28	10.82	10.86	10.5	10.67	0.116	0.809
	12	12.58	13.21	13.07	13.1	14.12	13.23	0.163	0.14
	24	16.56	15.14	16.46	16.21	16.21	16.43	0.239	0.605
Valeric acid	2	0.72	0.75	0.85	0.74	0.75	0.75	0.026	0.556
	4	1.04 ^a^	0.89 ^abc^	0.74 ^bc^	0.67 ^c^	1.00 ^ab^	0.68 ^c^	0.046	0.029
	8	0.93 ^ab^	0.93 ^ab^	0.67 ^c^	0.84 ^bc^	1.08 ^a^	0.92 ^ab^	0.038	0.03
	12	1.15	1.24	1.07	1.12	1.21	1.14	0.025	0.434
	24	1.16 ^b^	1.18 ^b^	1.15 ^b^	1.18 ^b^	1.37 ^a^	1.28	0.025	0.023
Isobutyric acid	2	0.38	0.36	0.4	0.39	0.38	0.38	0.004	0.28
	4	0.35 ^c^	0.37 ^bc^	0.50 ^a^	0.49 ^a^	0.45 ^ab^	0.45 ^ab^	0.017	0.005
	8	0.38 ^c^	0.42 ^bc^	0.58 ^abc^	0.8 ^a^	0.71 ^a^	0.67 ^ab^	0.046	0.018
	12	0.50 ^b^	0.56 ^b^	0.73 ^a^	0.76 ^a^	0.75 ^a^	0.70 ^a^	0.025	<0.001
	24	0.69 ^de^	0.59 ^e^	0.74 ^cd^	0.94 ^a^	0.86 ^ab^	0.80 ^bc^	0.027	<0.001
Isovaleric acid	2	0.47	0.44	0.52	0.42	0.47	0.4	0.015	0.198
	4	0.57	0.5	0.42	0.38	0.57	0.41	0.026	0.11
	8	0.53	0.54	0.46	0.55	0.62	0.55	0.023	0.396
	12	0.72	0.78	0.73	0.76	0.77	0.72	0.016	0.871
	24	1.01	0.96	0.82	1	0.96	1.03	0.023	0.072

^1^ CON: Control group; LD: Low-nitrogen group; LVA: LD group + 0.25% L-Val; LVB: LD group + 0.5% L-Val; LVC: LD group + 0.75% L-Val; LVD: LD group + 1% L-Val. ^2^ SEM, standard error of the mean. ^a–e^ indicate significant differences between values in the same row at *p* < 0.05.

**Table 7:** Effect of L-Val supplementation in low-protein diets on TVFA and acetate/propionate in vitro rumen fermentation fluid.

Item	Time/h	Treatment ^1^						SEM ^2^	*p*
		CON	LD	LVA	LVB	LVC	LVD		
TVFA/mmoL/L	2	58.83	58.55	60.51	59.43	60.34	59.59	0.247	0.106
	4	68.69 ^b^	68.92 ^b^	70.20	71.64 ^a^	71.17 ^a^	72.22 ^a^	0.401	0.017
	8	73.72 ^d^	74.49 ^cd^	74.66 ^bcd^	76.99 ^a^	76.77 ^ab^	76.49 ^abc^	0.381	0.015
	12	87.37 ^c^	87.97 ^bc^	90.44 ^ab^	90.36 ^ab^	91.20 ^a^	90.14 ^ab^	0.434	0.028
	24	101.90 ^bc^	100.80 ^c^	104.70 ^abc^	106.40 ^a^	105.50 ^ab^	106.20 ^a^	0.669	0.033
acetate:propionate	2	3.31	3.09	3.12	3.18	3.17	3.12	0.032	0.472
	4	3.18	3.07	3.14	3.00	3.13	3.15	0.033	0.745
	8	3.29	3.06	3.23	3.33	3.16	3.21	0.037	0.339
	12	3.47	3.38	3.41	3.52	3.64	3.59	0.035	0.211
	24	2.94	2.92	2.88	2.77	2.91	2.87	0.041	0.902

^1^ CON: Control group; LD: Low-nitrogen group; LVA: LD group + 0.25% L-Val; LVB: LD group + 0.5% L-Val; LVC: LD group + 0.75% L-Val; LVD: LD group + 1% L-Val. ^2^ SEM, standard error of the mean. ^a–d^ indicate significant differences between values in the same row at *p* < 0.05.

**Table 8:** Effects of L-Val supplementation in low-nitrogen diets on rumen CH_4_ production in vitro (mmol/mol).

Time/h	Treatment ^1^						SEM ^2^	*p*
	CON	LD	LVA	LVB	LVC	LVD		
2	293.51	283.3	284.4	287.51	287.66	284.82	1.169	0.110
4	284.31	280.76	283.7	278.6	281.44	285.02	1.165	0.651
8	290.92	282.05	289.8	291.21	284.15	286.86	1.296	0.200
12	296.11	293.44	294.2	296.88	301.35	299.57	1.001	0.148
24	279.97	277.55	277.8	271.2	277.41	276.09	1.71	0.832

^1^ CON: Control group; LD: Low-nitrogen group; LVA: LD group + 0.25% L-Val; LVB: LD group + 0.5% L-Val; LVC: LD group + 0.75% L-Val; LVD: LD group + 1% L-Val. ^2^ SEM, standard error of the mean.

## Data Availability

The original contributions presented in this study are included in the article. Further inquiries can be directed to the corresponding author.

## Abbreviations

The following abbreviations are used in this manuscript:

MCP	microbial protein
VFAs	volatile fatty acids
NH_3_-N	Ammonia Nitrogen
CP	Crude Protein
EE	Ether extract
ADF	Acid detergent fiber
NDF	Neutral detergent fiber
ME	Metabolizable energy
DM	Dry Matter
CH_4_	Methane
SEM	Standard Error of the Mean
L-Val	L-Valine
Lys	lysine
Met	methionine
Try	tryptophan
Thr	threonine
BCAAs	branched-chain amino acids
mmol	millimole
mol	mole
mL	milliliter
PCR	Polymerase Chain Reaction
μL	microliter
DNA	deoxyribonucleic acid
Ct	Cycle Threshold
ANOVA	Analysis of Variance
mg	milligram
A:P	Acetate/propionate
TVFA	total volatile fatty acid
IVA	Isovaleric acid
IBA	Isobutyric acid
Fs	*Fibrobacter succinogenes*
Bf	*Butyrivibrio fibrisolvens*
Sb	*Streptococcus bovis*
Ra	*Ruminobacter amylophilus*
